# Plant-Based Dietary Practices and Socioeconomic Factors That Influence Anemia in India

**DOI:** 10.3390/nu13103538

**Published:** 2021-10-09

**Authors:** Rohil S. Bhatnagar, Olga I. Padilla-Zakour

**Affiliations:** 1 Department of Food Science, Cornell University, Ithaca, NY 14853, USA; rb664@cornell.edu; 2 Tata-Cornell Institute for Agriculture and Nutrition, Cornell University, Ithaca, NY 14853, USA

**Keywords:** anemia, diet, fortification, India, iron bioavailability, iron deficiency, management, socioeconomic status

## Abstract

While rates of malnutrition have declined over the last decade in India due to successful government interventions, the prevalence of anemia remains high. Staple foods provide almost 70% of the daily iron intake. As staple foods are a rich source of phytate, this ingested iron is poorly absorbed. Currently, 59% of children below 3 years of age, 50% of expectant mothers and 53% of women aged 15–19 years are anemic. The most common intervention strategy has been through the use of iron supplements. While the compliance has been low and supplies irregular, such high rates of anemia cannot be explained by iron deficiency alone. This review attempts to fit dietary and cooking practices, field-level diagnostics, cultural beliefs and constraints in implementation of management strategies into a larger picture scenario to offer insights as to why anemia continues to plague India. Since the rural Indian diet is predominantly vegetarian, we also review dietary factors that influence non-heme iron absorption. As a reference point, we also contrast anemia-related trends in India to the U.S.A. Thus, this review is an effort to convey a holistic evaluation while providing approaches to address this public health crisis.

## 1. Introduction

Vitamins and minerals, which are classified as micronutrients, are essential nutrients required in small amounts to ensure optimal health and functioning of the human body. Iron is one such micronutrient that serves many key biologic functions—such as gene regulation, DNA synthesis, immune function, cofactor for enzymes, neurotransmitter synthesis, oxidation/reduction reactions and other metabolic reactions [1]. A key function of iron is the synthesis of the oxygen transporting proteins, hemoglobin (Hb) and myoglobin. A shortage of iron depletes the body of Hb, resulting in adverse health outcomes.

Even though iron is one of the most abundant elements present in the earth’s crust, its deficiency is also one of the most frequent nutritional disorders [2]. Iron depletion occurs when the mobilizable iron stores in the body get exhausted, but there is sufficient iron supplied to the erythron to carry out red blood cell (RBC) production. Over longer periods of depletion, there is less iron available to maintain the iron balance, unavoidably causing a decline in the RBC count. This pathological condition is termed as iron deficiency anemia (IDA) [3]. The most reliable clinical indicators to diagnose IDA are serum ferritin, serum total iron binding capacity and soluble transferrin receptor (sTfR) [4].

Currently, IDA affects over 1.2 billion people worldwide. It has been estimated that iron deficiency, without anemia, is at least twice as prevalent as IDA [5]. A recent data analysis by the World Health Organization (WHO) of 185 different countries from 1990 to 2011 showed that anemia has the highest prevalence and severity in pre-school children and women of reproductive age [6].

Several other etiologies have been implicated in the onset and progression of anemia. These include nutritional deficiencies in folate (B9), cobalamin (B12) and pyridoxine (B6); genetic disorders such as sickle cell anemia, thalassemia or aplastic anemia; acquired hemolytic anemia; anemia of inflammation or defective iron absorption due to celiac disease or other underlying etiologies [7,8]. Additionally, there is some evidence that vitamin A and D deficiencies may also contribute to anemia [9,10,11], but the exact mechanisms remain inconclusive.

The social and economic burden of IDA on developing countries is massive. In countries with a high prevalence of IDA, the productivity losses seem to be the greatest. It has been estimated that the impact of IDA on 6–59 months old children alone would account for USD 23 billion in total lifetime production losses, or a 1.3% loss of India’s GDP [12]. Although sustained efforts have been made to reduce the impact of iron deficiency through supplementation, fortification and biofortification programs, the prevalence of iron deficiency remains high [13].

As we will review in the following sections, IDA can have nutritional, physiological, pathological and (or) socioeconomic etiologies. Great efforts have been made to review one or few of these causal factors in-depth, hence providing only a partial perspective. However, to truly understand the magnitude of this nutritional issue, a more holistic outlook is warranted. Therefore, the objective of this article is to bridge varied disciplines in an attempt to offer a more complete evaluation of IDA, with a special focus on India. In the presented work, we examine dietary trends, cooking practices, environmental factors and societal norms that cause IDA in India, as well as past and current management strategies.

## 2. Iron Deficiency Anemia: The Indian Context

Even though India is self-sufficient in food production, it still suffers from high rates of undernutrition [14]. According to the fourth round of the National Family Health Survey (NFHS) in 2015–2016, anemia affects 59% of children below the age of 3 years, 50% of expectant mothers and 53% of women aged between 15 to 49 years in India [15]. This represents a minor improvement from the data collected during the third round of this survey in 2005–2006 [16]. However, across groups, this improvement was observed in the severe cases of anemia, which could also be a function of an increase in population size. In contrast, there was a 2% increase in children affected by mild anemia from 2005–2006 to 2015–2016. The rates of anemia in women have also increased from 2005–2006 to 2012–2014, with Uttar Pradesh showing a 40% rise in anemia prevalence [17]. In general, the prevalence of anemia is higher in poorer areas, socioeconomically lagging communities (scheduled castes and scheduled tribes) and among children born to uneducated women [16]. Despite the intervention strategies and a sustained rapid economic growth, IDA remains at the forefront of the public health catastrophes that plague India today. 

Although it is difficult to predict the number of anemic cases caused due to iron deficiency, it is estimated to be responsible for 25–60% of anemia [18,19]. Specifically in India, over half of the anemia burden can be attributed to dietary iron deficiency [13]. In South Asian countries including India, maternal anemia accounted for 18% of perinatal mortality and 20% of maternal mortality [20]. According to the Indian Council of Medical Research (ICMR), dietary iron deficiency contributed to 11% of all disability in India in 2016 [21].

### 2.1. Dietary Trends

**Figure 1:**
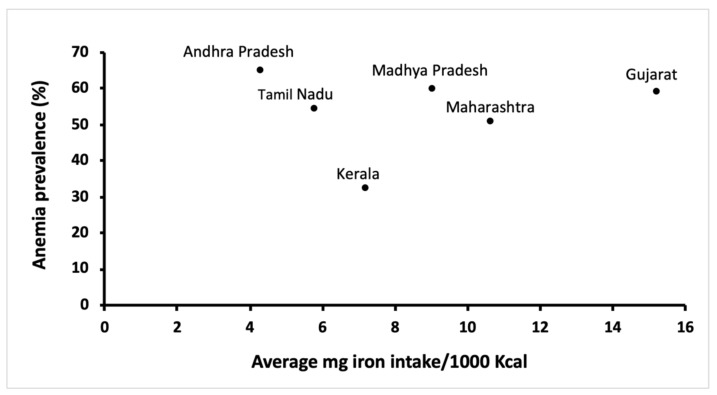
Iron consumption and anemia prevalence in rural women in six states of India [16,44].

According to Edelstein, India has more vegetarians than the rest of the world combined [22]. While this approximation may seem like an exaggeration, about 29% Indians aged 15 years and above are vegetarians [23]. Around 83% of those vegetarians adhere to a lactovegetarian dietary pattern, forgoing all animal-derived products except for dairy, 11% are lacto-ovo vegetarians (no meat, dairy and eggs are acceptable) and the remaining are vegans (dairy products prohibited) [24]. Dietary iron in foods may be categorized as one of two forms: inorganic iron (non-heme, primarily derived from vegetarian food sources, but also present in animal products) or organic iron (heme, derived from hemoglobin and myoglobin in meat) [25]. Meat-based heme iron has a much higher absorption (up to 35%) when compared to plant-based non-heme iron (2–10%) [4]. Moreover, meat can also enhance the absorption of non-heme iron [26,27]. However, in Indian diets, up to 95% of the total daily iron intake is through non-heme iron [28]. The main dietary iron sources in a vegetarian diet are legumes, dried beans, millets, soybean, nuts, dried fruits and green leafy vegetables [29]. Even though vegetarian diets may contain iron amounts comparable to omnivorous diets [30], the bioavailability of plant-based iron is impacted due to the presence of intrinsic factors that hinder absorption. Moreover, the bioavailability of plant-based iron can also be negatively affected upon co-consumption with other inhibitory foods. Since a routine meal may include several promoters and inhibitors of iron absorption, the net effect of these interactions on iron bioavailability can be difficult to predict [31], and this trend is clearly evident in Figure 1. Kerala has a predominant non-vegetarian population (97%), while over 60% of the population in Gujarat are vegetarians [23]. Likely, the consumption of animal flesh foods containing highly bioavailable iron resulted in a lower anemia prevalence in the Keralite population. Therefore, the quality or form of dietary iron is of crucial consideration. These results may also highlight the need to evaluate the relative contribution of other anemic etiologies, as will be reviewed in later sections. Wheat and polished rice form an integral part of the Indian dietary framework [32]. In 2011–2012, the bottom 25% of the Indian population received approximately 75% of their daily calories from staple cereals, while only 95 calories per day came from the consumption of fruits and vegetables [33]. As a result, the majority of the iron consumed per day was through staple cereal consumption. Meanwhile, the share of staple cereal in total calorie intake has witnessed a decrease in the past few years. However, this calorie deficit seems to have been met with an increased consumption of oil and sugar, while the share of fruits and vegetables or livestock has remained unchanged during this period [17]. Lastly, meat consumption in India is at 3.3 kg/capita, which is much lower than the world average of 34.3 kg/capita [34]. The resulting imbalance between requirements, intake and absorption through such limiting diets may predispose individuals to iron deficiency anemia. In contrast, while vegetarians may have lower iron stores than non-vegetarians [35,36,37,38,39], Hb levels of healthy vegetarians tend to be similar to non-vegetarians [40,41,42,43], suggesting that a balanced and well-planned vegetarian diet with iron coming from diverse food sources to promote adequate iron stores can offset lower Fe bioavailability.

To counterbalance low iron bioavailability, the U.S. Institute of Medicine (IOM) suggested that vegetarians should consume 80% more dietary iron [3]. Although in the right direction, this strategy might not be an immediately achievable solution with respect to India, where 89% of the population still consume an iron-deficient diet [45]. Due to a predominantly non-heme dietary pattern, poor iron bioavailability (50% lower) and Indian population characteristics, the ICMR has set higher levels of recommended dietary allowance (RDA) for iron for Indians, in comparison to the U.S. recommendations (Table S1, Supplementary Materials) [46].

Historically, adoption of the green revolution induced a shift towards maximizing the output of major staple crops—wheat, rice and maize. This caused two events: (1) reduced prices of these staple cereals [47], and (2) a decline in the cultivation of traditional nutrient-dense crops such as millets and pulses [48]. In many cases, this led people to consume more staples to meet their caloric demand and drastically diminished the dietary diversity [48], further enabling iron deficiency [49]. Such ramifications could be attributed, partially, to India’s food policy to favor caloric sufficiency over a nutritionally diversified food system [17].

### 2.2. The Contribution of Cooking Practices to Iron Deficiency Anemia in India

The past few decades have seen changes in cooking and food preparation methods in India. The use of traditional iron pots has been disregarded in favor of lighter, more aesthetic and easier-to-clean aluminum and stainless steel cooking vessels. As iron leaches into the food when cooked in cast iron pots [50,51,52,53,54,55,56], the transition away from it may have contributed to the rise in IDA [52]. Indeed, the beneficial effects from foods prepared in cast iron pots on iron status has been well documented [51,57,58,59]. Foods with an acidic pH tend to promote iron release from the vessel [60]. In contrast, a randomized control study on 6–59 months old infants and their mothers in Tanzania found no evidence that using stainless steel pots improved their iron status [61]. A study found that cooking traditional Indian chicken curry in iron karahi (Indian wok) improved the iron content fivefold over stainless steel cookware, while a staggering 27-fold increase was observed when cooking acidic (pH 4.14) tomato chutney [62]. The iron content of Indian snacks was enhanced by 16.2% when fried in a cast iron pot [51]. Kumari et al. also found a threefold increase when cooking green leafy vegetables in iron utensils when compared with steel or aluminum utensils [63]. While the benefits of cooking in iron pots is ancient knowledge, its popularity has not picked up despite the wide prevalence of IDA. One of the main reasons for the low compliance could be because cast iron vessels may rust and render a certain taste and color to the food, which may make it undesirable. This issue is aggravated when food is stored overnight in pots or when the pot is soaked in water for a longer duration to enhance the cleaning process [64]. In Ethiopia, it was found that smaller cast iron pots were generally more acceptable than larger pots [65]. Nonetheless, the use of cast iron pots is a feasible and inexpensive way to improve the intake level of bioaccessible iron. 

In addition, around 90% of rural Indian households and 32% of urban Indian households use biomass (dung, wood, straw, crop residue, charcoal) or fossil fuels (coal, kerosene) as an energy source [66]. The smoke emitted from the use of such fuels has been shown to elevate the risk of moderate to severe anemia (relative risk 1.79, 95% confidence interval 1.53–2.09) in pregnant women in Nagpur, India [67], likely due to the inflammatory mechanisms associated with chronic smoke inhalation [68].

### 2.3. Socioeconomic Etiologies

Socioeconomic self-explanatory determinants include joblessness, poor wages, poor sanitation and poverty, which may play a significant role in promoting IDA [69]. Irrespective of the household food security, certain traditional norms, such as “women eating last”, may have aggravated food insecurity amongst this demographic and pushed them towards IDA [70]. Most recently, Soumya et al. exhibited that empowering women in agricultural activities in Maharashtra enhanced their iron status, likely due to improved intrahousehold access to nutritional food [71]. It is important to highlight the role of sanitation as a cause of anemia in India. India has the highest number of open defecation-practicing population in the world [72]. The pathogens in the fecal matter can spread through contact. Amongst other adverse outcomes, microbial ingestion can decrease iron absorption through inflammatory pathways, as described in Section 4.3. In fact, anemia of inflammation (AI, also called anemia of chronic disease) is believed to be the second most common form of anemia globally [73].

### 2.4. Consequences of Iron Deficiency in Pregnancy

As per the ICMR, the RDA values for iron ranges from 17 mg for adult males, 21 mg for non-pregnant, non-lactating females and up to 35 mg for pregnant females [28]. Therefore, the demand on iron uptake for pregnant females is crucial. Clinical manifestations arising from iron deficiency anemia include pallor, fatigue, reduced working capacity and restless legs syndrome [74]. Due to the increased requirements for iron and smaller liver iron reserves, pregnant or menstruating women are at prime risk of developing this nutritional deficiency [75]. In fact, with every 1 mL of blood loss, there is a corresponding loss of 0.5 mg iron [76]. The maternal iron amount to cover pregnancy and childbirth in a 55 kg woman can be over 1200 mg [75]. Most of this iron transfer from the mother to the fetus occurs during the third trimester of gestation [77]. Since a considerable amount of placental blood transfusion takes places during the first few minutes after birth, premature umbilical cord clamping may promote iron deficiency in infants, especially in those born to anemic mothers [78]. Delayed cord clamping by 2–3 min can provide the infant with up to 24–32 mL of blood/kg body weight [79], equivalent to 75 mg of iron [80]. While immediate cord clamping is the common practice in India [81], the Government of India has recommended delayed cord clamping under the Anemia Mukt Bharat program instituted in 2018 to improve the iron status of infants [82]. It has been estimated that maternal iron deficiency anemia affects up to 50% of pregnancies [83,84], and not only predisposes the fetus to anemia, but also puts the mother’s life at great risk [85]. Accumulating evidence suggests that iron-deficient anemic women have shorter pregnancy durations than non-anemic women [86,87]. This can result in low birth weights and complications, including overall elevated maternal and neonatal mortality, caused by intrauterine growth retardation and pre-term delivery [88]. In this context, frequent blood testing in neonates lowers their iron status and increases the risk of IDA. Under iron deficiency, iron delivery is prioritized to meet the erythropoietic demands of the neonate rather than brain development [89]. Underdevelopment of the brain between 6–24 months of infant age cannot be reversed with iron supplementation later in life [90] and can lead to impaired cognitive function and delayed physical growth in children. It is noteworthy to mention here that traditional iron biomarkers measure only neonatal erythropoietic function, but there are currently no indicators to test for brain iron status [91]. As a result, brain iron deficiency may go undiagnosed and present adverse effects later. An additional condition that may enhance iron deficiency anemia is frequent pregnancy [3]. Given that the mother would have already spent over 1 g of her iron stores in her previous pregnancy, it is advisable that a sufficient time gap be given to the body to reaccumulate iron needed for her well-being, lactation (0.5–0.7 mg iron/d through breast milk) [92,93] and for the requirements of a subsequent pregnancy. Although it is recommended to have a birth spacing of at least 24 months, 27% of births in India occurred within 24 months of the previous birth [15]. IDA has severe consequences for the future generation, and without proper nutritional intervention, can eventually lead to a “double-burdened” society, with both the mother and the child being anemic.

### 2.5. The Contribution of Infant Complementary Feeding Practices to Iron Deficiency Anemia in India

Breast milk not only nourishes the infant, but also populates the infant gut microbiome and provides immunoglobulins that confer immunity against pathogens [94]. The nutrient requirements of the infant below 6 months of age can be met by adequate feeding of exclusive breast milk, provided that the mother and infant are in good health [95]. It has been estimated that the iron RDA of the infant is 0.23 mg/day [96]. Usually, the iron stores of the infant at birth are sufficient to last until 6 months. For infants born pre-term, these stores may be smaller and iron supplementation in the form of medicinal drops should be implemented at around 2–3 months of age to prevent iron deficiency anemia [97]. After 6 months of age, breast milk alone cannot meet the iron requirements and the infant should be introduced to optimal complementary foods to ensure proper growth and development. While breast milk remains an important nutrient source for the infant, and should be continued until 2 years of age, the WHO suggests that infants should be fed semi-solid foods rich in iron, and usually some meat products [95].

In India, it is recommended to feed the infant a thick porridge made from staples such as semolina, broken wheat, wheat flour or millet. Other foods could include lentils, mashed fruits or iron-fortified instant food mixes [98]. However, these cereal-based complementary foods suffer from low iron bioavailability and may defeat the purpose altogether. The minimum acceptable diet criterion is a function of minimum dietary diversity and minimum feeding frequency, set forth by the WHO [95]. Minimum dietary diversity is explained as consuming at least four food groups while meeting the minimum meal frequency. These food groups are grains, roots and tubers; legumes and nuts; dairy; flesh foods; eggs; vitamin A-rich food and vegetables; other fruits and vegetables [95]. In the most recent NFHS survey in India, only 4.9% of children aged 6–8 months met this criterion [15]. This survey also collected dietary information on iron-rich food intake based on a 24 h recall period. Only about 18% of children aged 6–59 months consumed iron-rich foods on the previous day of the survey, while 20.7% of non-breastfed children in the 6–23 months age group consumed fortified baby foods. Consequently, only 8.7% of breastfeeding children (rural and urban) aged 6–23 months received a minimum acceptable diet. 

### 2.6. The Relationship between Iron Deficiency and Lead Toxicity

Lead (Pb^2+^) is a toxic heavy metal that can adversely affect cognitive development in children [99]. While lead toxicity affects children across sociodemographics, children belonging to the lower socioeconomic status present the highest burden. Children belonging to poor families are more likely to reside in sub-standard housing with lead-based paint, or in areas near industrial waste or a landfill. Lead exposure can be substantial in such children [100]. As anemia is widely prevalent in this age group [15], it can aggravate lead toxicity. This is because under chronic iron deficiency, iron transporter divalent metal transporter-1 (DMT1) becomes more efficient at absorbing divalent metals [101], and due to the frequent hand-to-mouth activity in children, can result in lead poisoning [102]. A high amount of blood lead levels (>10 μg/dL) have been reported in Indian children [103,104,105]. Since lead in gasoline was also a contributing factor towards lead toxicity in children, its removal from industrial gasoline production is reported to have decreased blood lead concentrations [106,107]. In such children, iron fortification was found to reduce blood lead concentrations [105], likely due to the higher affinity of DMT1 for iron as compared to lead. Consequently, this would have improved the child’s iron status and in turn reduced the DMT1 activity.

### 2.7. Laboratory Diagnostic Biomarkers and On-Field Practices

Generally, Hb determination is employed to detect anemia in rural areas in India, where facilities to conduct more specialized tests are often lacking [15]. As such, Hb testing is a non-specific marker to determine IDA. In rural areas with poor water, sanitation and hygiene (WASH) practices, it may be most important to differentiate between IDA and AI (Table 1). As discussed earlier, severely depleted body iron stores lead to IDA. In contrast, iron is sequestered in ferritin under AI. Therefore, serum ferritin is a widely used measurement, with values lower than 15 μg/L indicative of IDA, and < 30 μg/L as diagnostic for IDA in the presence of infection [108]. It is important to note that IDA and inflammation may coexist. The accurate assessment of IDA, thus, requires multiple test results. Certain acute-phase proteins, such as C-reactive protein and α1-acid glycoprotein, have been used in this regard. Since the spike in C-reactive protein levels is short-lived under inflammation, α1-acid glycoprotein is usually preferred, as these values tend to stay higher for longer, usually weeks, allowing for an easier diagnosis [109]. Under iron deficiency, sTfR is upregulated, while its values remain unaffected under inflammatory conditions [110,111]. Therefore, sTfR is a very useful biomarker to differentiate between the anemia of iron deficiency or anemia of chronic inflammation [112]. A combination of these indicators provides a simple and reliable diagnosis of iron deficiency in populations [113]. Other definitive and well-established biochemical measurements that can be used to detect IDA are zinc protoporphyrin levels, transferrin saturation and reticulocyte hemoglobin.

To alleviate anemia symptoms, most interventions focus on providing high-dose iron supplementation to at-risk populations (as outlined in the subsequent section). While iron supplementation has proven beneficial in clearly iron-deficient populations [116,117,118,119,120,121,122], it has been detrimental in communities with high infection rates and/or in iron-replete individuals [123,124]. It has been observed that supplementing iron in the form of micronutrient powders in malaria-endemic areas increased intestinal inflammation and enhanced the abundance of enteric pathogens in the gut microbiome of anemic infants in Kenya [125]; increased the rates of hospital admissions (relative risk 1.11, 95% confidence interval 1.01–1.23) and mortality (relative risk 1.16, 95% confidence interval 0.92–1.47) in children aged 1–35 months in Pemba, Zanzibar [126] and caused gastrointestinal (G.I.) distress and bloody diarrhea (relative risk 1.63, 95% confidence interval 1.12–2.39) in 6–18 months old children in Pakistan [127]. Therefore, untargeted iron supplementation in high infection transmission areas may inadvertently worsen health outcomes, and it is advisable to proceed with caution when implementing universal iron supplementation programs.

## 3. Management Strategies

### 3.1. Government Iron Supplementation Programs

The Government of India has initiated several key programs in the past to combat the burden of IDA in India (Table S2, Supplementary Materials). However, iron–folic acid (IFA) supplementation has been met with low compliance from the beneficiaries. Certain side effects, such as vomiting, mild nausea, gastritis, constipation and dark stool, have been attributed as reasons for forgoing IFA tablet use [128]. In 2001, the Supreme Court of India mandated the Indian government to provide hot cooked meals in government schools for 200 days per annum [129]. These mid-day meals have benefited over 120 million schoolchildren by providing a balanced and nutritious meal [130], and may have also ensured continuous school enrolment [131] and daily pupil attendance [132]. The most recent strategy by the Government of India and the Ministry of Health and Family Welfare is the Intensified National Iron Plus Initiative (I-NIPI) in 2018 with the motto “anemia mukt bharat” (anemia-free India). This initiative was conceived with an ambitious goal to reduce anemia by 50% among women of reproductive age (WRA) by 2025, and lower anemia rates in infants, adolescents and WRA by 3% points each year until 2020, covering 450 million beneficiaries. This multi-pronged program includes the provision of prophylactic IFA pills; deworming tablets; testing and treatment of anemic cases; iron- and FA- fortified foods through government public health programs; increasing awareness about anemia and its causes and including behavior change modules focused on compliance, deworming, infant feeding practices, improving dietary diversity and intake of iron-rich foods and delaying cord clamping during birth by 3 min [82].

### 3.2. Food-Based Approaches

Food fortification is an inexpensive, effective and scalable public health solution that has been successfully implemented in many countries to increase dietary iron intake [133]. The Copenhagen Consensus expert panel ranked iron fortification as the second highest development priority [134]. According to the WHO, if iron-fortified foods were available to 50% of the population in the Southeast Asian sub-region (including India), it will save an estimated 587,052 disability-adjusted life years (DALYs), resulting in USD 43 per DALY averted [135]. India has previously seen success with mandatory fortification programs. With mandatory iodine fortification of salt since 1998, 93.1% households have reported consuming iodine fortified salt on a daily basis [15]. In fact, the sale of non-iodized salt for human consumption is banned in India [136]. 

Due to their mass consumption in areas where plant-based diets and anemia predominate, cereal flours and rice are two of the most commonly iron fortified food vehicles [137,138]. Almost 65% of the Indian population consider rice as a food staple, while the average consumption of wheat flour in Indian adults has been estimated to be around 150–200 g per day [139]. Clinical studies on iron fortification in salt [140,141], rice [105,119,142,143,144], wheat flour [117] and other foods [145,146,147] in Indian population have shown to improve Hb concentrations, serum ferritin levels and alleviate iron deficiency anemia. In 2016, the Food Safety and Standards Authority of India (FSSAI) adopted standards to fortify common staple foods such as wheat flour, rice and salt with iron. Fortified products are denoted with an F+ logo (Figure S1, Supplementary Materials) to differentiate them from unfortified foods. The recommended fortification rates for these three food staples have been included in Table 2. Incremental costs to fortify food staples with iron is minimal, ranging from 8 paise/kg flour (USD 0.001/kg) to 4 INR/kg salt (USD 0.054/kg). To maintain the quality and cost-effectiveness in the long term, iron-fortified wheat flour requires pre-packaging at a centralized facility with trained personnel. Such branded flours incur a 5% goods and sales tax (GST), levied by the government. In contrast, the unpackaged (and largely unfortified) flour obtained through the local village-level miller comprises 95% of the Indian market and is GST exempt. It can be a challenge for local millers to cover the initial capital costs associated with the purchase and maintenance of balances, feeders and blenders needed for fortification, and employing trained personnel to operate equipment. Moreover, the markets for commonly fortifiable foods are largely unorganized [148]. To overcome the above challenges for a successful fortification program, recommendations include: (1) central government financial assistance to cover the initial costs needed for fortification for small producers; (2) GST tax differential should be adequately addressed; (3) stringent monitoring programs should be implemented to ensure compliance and (4) a steady supply of iron-fortified foods in the market should be ensured.

Biofortification is a recent food-based strategy to enhance the concentrations of targeted micronutrients in staple food crops using conventional breeding and agronomic approaches [149]. Given that iron deficiency anemia afflicts poor people the most, biofortification can improve nutritional security in populations that predominantly rely on calorie-dense staples for nourishment, and where coverage concerns make continued food supply an issue. Moreover, most of the produce is consumed within local communities. This approach is self-sustaining as it only requires a one-time dissemination or purchase of seeds [150]. It has been estimated that the biofortification of rice and wheat alone could reduce the burden of iron deficiency in India by as much as 58% [151]. While there is currently no commercially available iron-biofortified food crop in India, randomized trials with iron-biofortified pearl millet have shown to improve indices of iron nutrition [152] and cognition [153] in children. However, the presence of inhibitors, such as phytic acid, may affect iron bioavailability, and remains a cause of concern. Nevertheless, biofortification as a supplemental approach holds tremendous promise in reducing the iron deficit gap with minimum research and implementation investment.

Enhancing dietary diversity is another approach to improve iron nutrition in vulnerable populations. This requires continuous access to varied foods from different food groups and in adequate quantities. Aside from increasing dietary iron intake, this approach may also improve the intake of other nutrients simultaneously. The National Nutrition Mission (Poshan Abhiyaan) is a new initiative by the Government of India to reduce malnutrition in children (6 months–6 years) and women (15–49 years) [154]. Amongst other nutrition strategies, food fortification and dietary diversification are key components of this mission [155]. In addition, knowledge dissemination on reviving traditional nutrient-rich food systems and behavior change practices are emphasized [156]. State missions are being encouraged to promote nutrition-sensitive integrated farming systems in order to enable the diversification of diets and ensure food availability throughout the year [157]. Despite improved access to iron-rich foods, poorer populations may have financial constraints to purchasing a wide range of foods, posing a major limitation.

## 4. Modulators of Iron Bioavailability

Bioavailability can be defined as the fraction of an ingested nutrient that is available to the body through absorption for utilization in normal physiological functions and for metabolic processes [158]. The amount of iron present in the diet and its relative bioavailability are the two most critical determinants in ensuring optimal iron nutrition [49]. Non-heme iron bioavailability is heavily influenced by meal composition. Once the iron in a meal is ingested, it enters a labile pool in the lumen of the gastrointestinal (G.I.) tract, where its absorption is dependent on the presence of various inhibitors or promoters [159].

### 4.1. Dietary Inhibitors of Iron Absorption

Phytic acid (myo-inositol hexaphosphoric acid) is a naturally occurring compound in cereal grains, legumes, oil seeds and nuts as the principal storage form of phosphorus. Concentrations of phytates in cereal and legume grains may be as high as 5% of the dry weight [160]. Phytic acid forms insoluble aggregates with iron in the G.I. lumen and makes it unavailable for absorption under the physiologic pH of the small intestine [161]. Certain food processing and preparation strategies, such as germination, malting, soaking and fermentation, can be effectively utilized to degrade phytate and release bound iron, primarily by improving the activity of the enzyme phytase that catalyzes the breakdown of phytate [162,163]. Interestingly, preparation of chapatis (form of Indian bread) from flour has been shown to increase phytic degradation by 40–50% [164,165]. Indians consume an estimated 1287–2500 mg of phytate per day [166,167,168]. To put that into perspective, an increase in phytate content from 0.3 g/d to 1.3 g/d in Indian regional diets decreased iron availability from 7.9% to 1.52% [169]. Therefore, such high daily intakes of phytate can have far-reaching consequences. The average phytate and iron content of common staple cereals are listed in Table 3.

Polyphenols are effective iron chelators [175] and similar to phytic acid, can also complex with iron. Since polyphenols are ubiquitous in most foods, their interaction with non-heme iron is unavoidable. Siegenberg et al. exhibited that even a minor inclusion of tannic acid (55 mg) in a bread meal reduced iron absorption by 67% in Indian women [176]. It is noteworthy to mention that not all polyphenols possess the same inhibitory effect on bioavailable iron. For the same amount of polyphenolic content, black tea polyphenols had a sharper inhibitory effect than red wine, herbal tea and coffee polyphenols [177,178]. India is the largest consumer of black tea in the world with the consumption of over one billion kilograms in 2020 alone [179]. This may play a major contribution towards IDA in India, especially since the intake of black tea with milk (as is the regular consumption practice in India) has been shown to have a larger inhibitory effect on iron absorption than tea alone [180]. Spices are rich in polyphenols and abundantly consumed in India. Tuntipopipat et al. showed that except for tamarind, all of the spices tested (chili pepper, garlic, shallots and turmeric) decreased the amount of dialyzable iron in a dose-dependent manner in vitro [181]. In contrast, while chili inhibited iron absorption in female subjects, turmeric had no effect [182].

Even though some vegetables are rich sources of iron, their bioavailability is severely impacted due to the presence of iron inhibitors, such as oxalate. Commonly consumed Indian vegetables were reported to contain high amounts of oxalate, ranging from 5 g/100 g DM in Colocasia leaves to over 12.5 g/100 g DM in spinach [183].

### 4.2. Dietary Promoters of Iron Absorption

Considering a vegetarian dietary pattern, ascorbic acid (AA, vitamin C) is a strong enhancer of non-heme iron absorption [184]. The potentiating effect of AA is primarily attributed to its ability to reduce Fe^3+^ to Fe^2+^, but it is also a potent iron chelator [185]. In fact, AA can successfully negate the inhibitory effects of both polyphenols [186] and phytates [187,188]. Supplementation of ascorbic acid to Indian meals have shown to significantly improve iron status [189,190,191,192]. However, the average Indian intake of vitamin C has been reported to be very low [169]. Onions and garlic, alone [193] or in combination with dried mango powder (amchur), and β-carotene rich vegetables [194] have also been shown to produce synergistic improvements in the amount of bioaccessible iron through cereal and pulses in an Indian meal. 

### 4.3. Influence of Physiologic Factors on Iron Absorption

Besides the dietary composition, the subjects’ physiologic conditions also influence non-heme iron absorption. The iron requirement is greater during pregnancy [77], iron deficiency [195], adolescence and menstruation. In such situations, the body tends to absorb more iron from inorganic sources to maintain an iron balance. The need for iron is escalated in pubertal girls, as on one hand they require iron to accommodate the increase in blood volume associated with a growth spurt, while they also begin to undergo iron losses due to menstruation [196,197]. Anemia is often observed under clinical cases of infection or inflammation, termed as anemia of inflammation. Much like for humans, iron is an essential growth nutrient for pathogens. As a stress response to infection, the body withholds iron from pathogens by releasing the hepatic hormone hepcidin, thereby (1) inhibiting iron efflux from enterocytes and macrophages via the degradation of iron exporter ferroportin, and (2) limiting the release of stored iron from hepatocytes [198]. Recent studies have exhibited the putative role of the cytokine interleukin-6 in stimulating hepcidin production [199,200]. These events limit iron availability to pathogens. In fact, under inflammatory conditions, plasma ferritin can increase by as much as 300% [201].

## 5. Conclusions

Despite iron supplementation programs in place, anemia has continued to prevail, especially amongst the vulnerable. Hb quantification is the main screening test to detect anemia. Since anemia is multi-factorial and excess iron can be detrimental to health, iron studies at the field level will be beneficial in order to adequately determine and address the etiology of anemia. While technical limitations at the field level continue to impede a specific diagnosis, scientific advances and new rapid testing may make point-of-care hematology more accessible. It remains to be seen when such clinical capabilities can be effectively implemented.

Given that rural areas may have high infection rates, it may be prudent to approach untargeted iron supplementation with caution as it may cause more detriment than benefit. In clearly iron-deficient populations, there should be continued availability of IFA tablets. Monitoring programs should be in place to assess the compliance, and regular supervision is advisable to ensure that monitoring is carried out effectively. Food-based approaches may be the most acceptable method to improve iron intakes in such populations. Because food fortification programs require effective coverage, accessibility and equitable distribution to be successful, complementary practices that can be easily performed at the household level, such as healthy and balanced food preparation to reduce phytate content in foods, co-consumption of meals with iron promoters or cooking in cast iron pots, should be encouraged. Iron-rich traditional hardy crops, such as pearl millet and sorghum, can improve the iron status and health of at-risk communities. Biofortification can further enhance the iron content in these cereals. Looking ahead, the ability of biofortification to make a nutritionally significant impact on the iron status in India is dependent on collaborative efforts between the policy makers, agricultural research institutes, food and seed companies and farmers.

For the above interventions to be successful, there is a strong need to increase education on sanitary guidelines and implementable strategies to improve nutrition. Only under such circumstances can diet-based interventions yield desirable results.

## Figures and Tables

**Table 1:** Contrasting diagnosis of iron-deficiency anemia and anemia of inflammation *.

	Iron-Deficiency Anemia	Anemia of Inflammation
Anemia classification	Microcytic	Normocytic
Hemoglobin	↓	↓
Serum iron	↓	↓
Ferritin	↓	↑
Transferrin saturation	↓	↓—R
Total iron binding capacity	↑	↓
Mean corpuscular volume	↓	↓—R
Soluble transferrin receptor	↑	↓—R
Soluble transferrin receptor to ferritin ratio	↑	R
Erythrocyte zinc protoporphyrin	↑	↑
Reticulocyte hemoglobin	↓	↓
Hepcidin	↓	↑

* Arrows denote the direction of change, R = reference values [113,114,115].

**Table 2:** Iron fortification standards recommendations by the Food Safety and Standards Authority of India.

Staple	Iron Source	Fortification Level (per Kg)
Rice	Sodium iron (III) ethylenediaminetetraacetate trihydrate (Na Fe EDTA) Ferric pyrophosphate	14–21.25 mg 28–42.50 mg
Atta (wheat)	Sodium iron (III) ethylenediaminetetraacetate trihydrate (Na Fe EDTA) Iron–Ferrous citrate or ferrous lactate or ferrous sulfate or ferric pyrophosphate or electrolytic iron or ferrous fumarate or Ferrous bisglycinate	14–21.25 mg 28–42.50 mg
Salt	Ferrous sulfate or ferrous fumarate	850–1100 mg

Source: [148].

**Table 3:** Typical iron and phytic acid content in commonly consumed Indian staple foods.

Staple Foods	Iron Content (mg/100 g dw)		Phytic Acid Content (g/100 g dw)		
	Whole Grain	Milled	Milled	Soaked (12–16 h)	Germinated (24 h)
Sorghum	7.65	3.95	1.08	1.09	1.08
Soybean	7.19	3.63	1.40	1.36	1.44
Kidney bean	8.60	6.30	1.08	1.21	1.22
Millet	11.10	6.42	0.83	0.72	0.71
Cowpea	6.60	5.50	0.66	0.61	0.66
Green gram	4.89	3.93	0.83	0.89	0.79
Lentil	7.57	7.06	1.15	1.12	1.12
Chickpea	6.78	6.08	0.48	0.41	0.51
Black gram	5.97	4.67	0.65	0.44	0.44
Pigeon pea	5.37	3.90	0.63	0.62	0.57
Barley	5.00	1.56	1.01	0.95	0.86
Wheat	3.97	1.77	1.03	1.04	1.12
Maize	2.49	1.10	1.15	1.14	1.16
Rice	1.02	0.65	0.88	0.62	0.53

Source: iron content: [170,171,172,173]; phytic acid: [162,174].

## Supplementary Materials

The following are available online at https://www.mdpi.com/article/10.3390/nu13103538/s1, Figure S1: Fortification logo used in India., Table S1: Recommended dietary allowances (RDA) for iron for Indian Council of Medical Research (ICMR) as compared to U.S. Institute of Medicine (IOM) recommendations (mg/d), Table S2: Previous iron and folic acid (IFA) supplementation programs in India.
