# Supplementary material for: Plant-Based Dietary Practices and Socioeconomic Factors That Influence Anemia in India
Source: Nutrients. 2021 Oct 9;13(10):3538. doi: 10.3390/nu13103538 (PMC8537570; doi:10.3390/nu13103538)
Supplement: Supplementary file 1 [file nutrients-13-03538-s001.zip › nutrients-1377623-supplementary.pdf]

Supplemental File 1

Table S1. Recommended dietary allowances (RDA) for iron for Indian Council of Medical Research (ICMR) as compared to U.S. *Institute of Medicine (IOM) recommendations (mg/d)*

**Group**	**Age**		**RDA (ICMR)**	**RDA (IOM)**
Infants	0 – 6 mo*		0.27	0.27
	7 – 12 mo		5	11
Children	1 – 3 y		9	7
	4 – 6 y		13	10
	7 – 8 y		16	10
	8 – 9 y		16	10
Adolescents	9 – 10 y	Boys	16	8
		Girls	16	8
	10 – 12 y	Boys	21	8
		Girls	27	8
	12 – 13 y	Boys	21	8
		Girls	27	8
	13 – 14 y	Boys	32	8
		Girls	27	8
	14 – 15 y	Boys	32	11
		Girls	27	15
	16 –18 y	Boys	28	11
		Girls	26	15
Adults	Male		17	8
	Female	Non-pregnant, non-lactating	21	18
		Pregnant	35	27
		Lactating	25	9

*There is no RDA for infants aged 0 – 6 months. As exclusive breast milk feeding is expected to meet iron requirements at this age, the average intake is set at 0.27 mg/d based on the amount of iron present in breast milk (0.3 mg/L) and the mean volume of breast milk consumed by the infant (0.9 L).

Source : [1,2]

Table S2. Previous iron and folic acid (IFA) supplementation programs in India

Year	Program	Agenda	Limitations	References
1970	National Nutritional Anemia Prophylaxis program	Women (pregnant/lactating) provided with IFA tablets containing 60 mg elemental iron and 500 µg folic acid per tablet daily for 100 days; pre-school/young children received liquid supplements containing 20 mg elemental iron and 100 µg folic acid for 100 days per year	65% of the women received, or purchased IFA tablet, but only 23% fulfilled the entire dosage period; iron contents of 30% tablets were found to be lower than prescribed, while none of the tablets matched the recommended folic acid levels; irregular supplies and poor coverage	[3,4]
1991	National Nutritional Anemia Control program	All women (anemic or non-anemic, 100 mg elemental iron) and children received IFA tablets, along with a nutritional message to increase intake of iron-rich foods	This intervention was limited by irregular supplies, poor compliance and low nutritional counseling	[5–7]
2007	12 × 12 initiative	Provision of IFA supplements and nutritional education to adolescents, along with deworming tablets and immunizations; the goal was to achieve Hb levels of 12 g/dL for all adolescents by 12 years of age by 2012	Poor knowledge about nutritional causes of anemia, inadequate supplies of pills, inadequate coverage, poor management of parasitic infection and inability to reach the target population thwarted its success	[8,9]
2013	National Iron Plus Initiative	The beneficiaries (>6 months old) received lifelong iron supplementation. Pre-school children (6–60 months old) received 1 mL of liquid IFA syrup containing 20 mg elemental Fe and 100 µg FA biweekly; children between 5–10 years of age received tablets containing 45 mg elemental Fe and 400 µg FA weekly; adolescents and women of	Operational issues, poor coverage; low compliance; iron supplementation strategies did not improve anemia prevalence as expected, perhaps other etiologies were overlooked	[10,11]

	reproductive age (WRA) received weekly supplementation tablets containing 100 mg Fe and 500 µg FA; and pregnant or lactating women received the same dose as WRA with increased frequency (a tablet per day for 100 days)	

Figure S1. Fortification logo used in India. "The square around the +F represents completeness. The plus sign is about adding extra nutrition via vitamins and minerals to the daily nutrition requirements. The ring illustrates the ring of good health, protection for an active and healthy lifestyle." [12]

References

1. Narasinga Rao, B.S.; Sivakumar, B. Nutrient Requirements & Recommended Dietary Allowances for Indians (1990, Reprinted 2008) 2nd Edition - 2010) By B.S. Narasinga Rao, B. Sivakumar) | Indian Council of Medical Research | Government of India; Nutrient Requirements & Recommended Dietary Allowances for Indians (1990, Reprinted 2008); 2nd ed.; Indian Council of Medical Research (ICMR), **2010**.
2. Institute of Medicine (U.S.); Panel on Micronutrients Dietary Reference Intakes for Vitamin A, Vitamin K, Arsenic, Boron, Chromium, Copper, Iodine, Iron, Manganese, Molybdenum, Nickel, Silicon, Vanadium, and Zinc; National Academy Press: Washington, D.C., **2002**; ISBN 978-0-309-07279-3.

3. International Institute for Population Sciences (IIPS) National Family Health Survey (NFHS-3) 2005-2006, Volume 1; Government of India: Mumbai:IIPS, **2007**.
4. Vijayaraghavan, K.; Brahmam, G.N.; Nair, K.M.; Akbar, D.; Rao, N.P. Evaluation of National Nutritional Anemia Prophylaxis Programme. Indian J Pediatr **1990**, 57, 183–190.
5. Ministry of Health & Family Welfare; Government of India Policy on Control of Nutritional Anaemia; **1991**; p. 11.
6. Deshmukh, P.R.; Garg, B.S.; Bharambe, M.S. Effectiveness of Weekly Supplementation of Iron to Control Anaemia Among Adolescent Girls of Nashik, Maharashtra, India. Journal of Health, Population and Nutrition **2008**, 26, 74–78.
7. Kotecha, P.V. Nutritional Anemia in Young Children with Focus on Asia and India. Indian J Community Med **2011**, 36, 8–16.
8. Upadhyay, R.; C, P.; Kulkarni, V.; Kulkarni, V. Unrelenting Burden of Anaemia in India: Highlighting Possible Prevention Strategies. International Journal of Medicine and Public Health **2012**, 2, 1–6.
9. Anand, T.; Rahi, M.; Sharma, P.; Ingle, G.K. Issues in Prevention of Iron Deficiency Anemia in India. Nutrition **2014**, 30, 764–770.
10. Kapil, U.; Kapil, R.; Gupta, A. National Iron Plus Initiative: Current Status & Future Strategy. Indian Journal of Medical Research **2019**, 150, 239.
11. Kapil, U.; Bhadoria, A.S. National Iron-plus Initiative Guidelines for Control of Iron Deficiency Anaemia in India, 2013. Natl Med J India **2014**, 27, 27–29.
12. Food Safety and Standards Authority of India Large-Scale Food Fortification in India: The Journey so Far and Road Ahead; **2017**.
